# Stress-induced DNA damage biomarkers: applications and limitations

**DOI:** 10.3389/fchem.2015.00035

**Published:** 2015-06-02

**Authors:** Zacharenia Nikitaki, Christine E. Hellweg, Alexandros G. Georgakilas, Jean-Luc Ravanat

**Affiliations:** ^1^DNA Damage and Repair Laboratory, Physics Department, School of Applied Mathematical and Physical Sciences, National Technical University of AthensAthens, Greece; ^2^Radiation Biology Department, German Aerospace Center (DLR), Institute of Aerospace MedicineKöln, Germany; ^3^Laboratoire des Lésions des Acides Nucléiques, Institut des Nanosciences et Cryogénie, Service de Chimie Inorgranique et Biologique, Université Grenoble AlpesGrenoble, France; ^4^CEA, Institut des Nanosciences et Cryogénie, Service de Chimie Inorgranique et BiologiqueGrenoble, France

**Keywords:** DNA damage, oxidative stress, biomarkers, ionizing radiation, UV radiation, clustered DNA lesions

## Abstract

A variety of environmental stresses like chemicals, UV and ionizing radiation and organism's endogenous processes such as replication stress and metabolism can lead to the generation of reactive oxygen and nitrogen species (ROS/RNS) that can attack cellular vital components like DNA, proteins and lipid membranes. Among them, much attention has been focused on DNA since DNA damage plays a role in several biological disorders and aging processes. Thus, DNA damage can be used as a biomarker in a reliable and accurate way to quantify for example radiation exposure and can indicate its possible long term effects and cancer risk. Based on the type of DNA lesions detected one can hypothesize on the most probable mechanisms involved in the formation of these lesions for example in the case of UV and ionizing radiation (e.g., X- or α-, γ-rays, energetic ions, neutrons). In this review we describe the most accepted chemical pathways for DNA damage induction and the different types of DNA lesions, i.e., single, complex DNA lesions etc. that can be used as DNA damage biomarkers. We critically compare DNA damage detection methods and their limitations. In addition, we suggest the use of DNA repair gene products as biomarkes for identification of different types of stresses i.e., radiation, oxidative, or replication stress, based on bioinformatic approaches and meta-analysis of literature data.

## Introduction

### DNA damage formation and consequences also including DNA repair

In all cells and tissues a significant level of DNA damage is formed on a daily basis from exposure to various intracellular and extracellular agents. Endogenous sources of damage targeting nuclear and mitochondrial DNA are but not limited to replication stress and oxidative stress i.e., free radicals resulting from metabolism as by-products and at the level of the organism from various mechanisms like inflammatory responses such as reactive oxygen species (ROS) released from macrophages (Kryston et al., 2011). In addition, the so-called oncogene-induced ROS can fuel high proliferation, replication stress and DNA damage response (DDR) activation (Ogrunc et al., 2014). Replication stress manifested as stalled replication forks and possible collapse results in DNA double strand breaks (DSBs) which must be repaired immediately usually by the homologous recombination (HR) pathway (Halazonetis et al., 2008). This continuous challenging process may lead to genomic instability and cancer, especially if it is combined with exposure for example to natural radiation and low doses of ionizing radiation from medical exams (X-rays). This “naturally” occurring combination of DNA damage (DSBs and oxidized bases) may be considered as the most common form of complex DNA damage, triggering different repair mechanisms in the cell, such as DSB repair (HR and non-homologous end joining-NHEJ), base excision repair (BER), mismatch repair (MMR), and possibly nucleotide excision repair (NER) especially for UV-induced DNA lesions (Aziz et al., 2012). In order to complete the picture one should add the fact that specific regions of the human genome are prone to breaks i.e., the fragile sites are highly preferable targets for DNA breakage due to replication or oxidative stress (Tsantoulis et al., 2008; Georgakilas et al., 2014). These problematic regions of possible DNA breaks do not relate necessarily with the genome sites where radiation interacts creating may be an accumulative phenomenon of genome “damage burden.” Last but not least, at the organism level, these phenomena maybe augmented by the initiation of systemic effects inducing DNA damage in distant sites of the human body as a result of innate or adaptive immune response (Sprung et al., 2015).

In this review we summarize the current knowledge of DNA damage induction mechanisms and the primary methodology utilized for detection and quantification of DNA lesions generated by a variety of stresses expected to induce the majority of DNA lesions in the cell. Although these classifications are introduced for the first time in this work in general there are considered as “classical” examples of DNA damage sources (Aziz et al., 2012). Since we consider the critical use of DNA damage-based biomarkers not only for biological dosimetry of radiation exposure but also for prediction of radiation effects and prognosis of cancer radiotherapy, we performed a meta-analysis of literature available data, to identify putative DDR genes implicated in the cellular reaction to three primary types of DNA damage inducing stresses: ionizing radiation, oxidative, and replication stress. Our results suggest some genes which are possibly “unique” for each type of stress and may be candidates for DNA damage biomarkers i.e., markers indicating that DNA damage has occurred and most possibly DDR initiation. The critical parameter here is that these markers are assigned in each case to three different types of “stress”: (1) ionizing radiation, (2) oxidative stress, and (3) replication stress.

## Formation of DNA damages: mechanistic aspects

### Non-ionizing radiation: UV induced DNA damage

**Figure 1:**
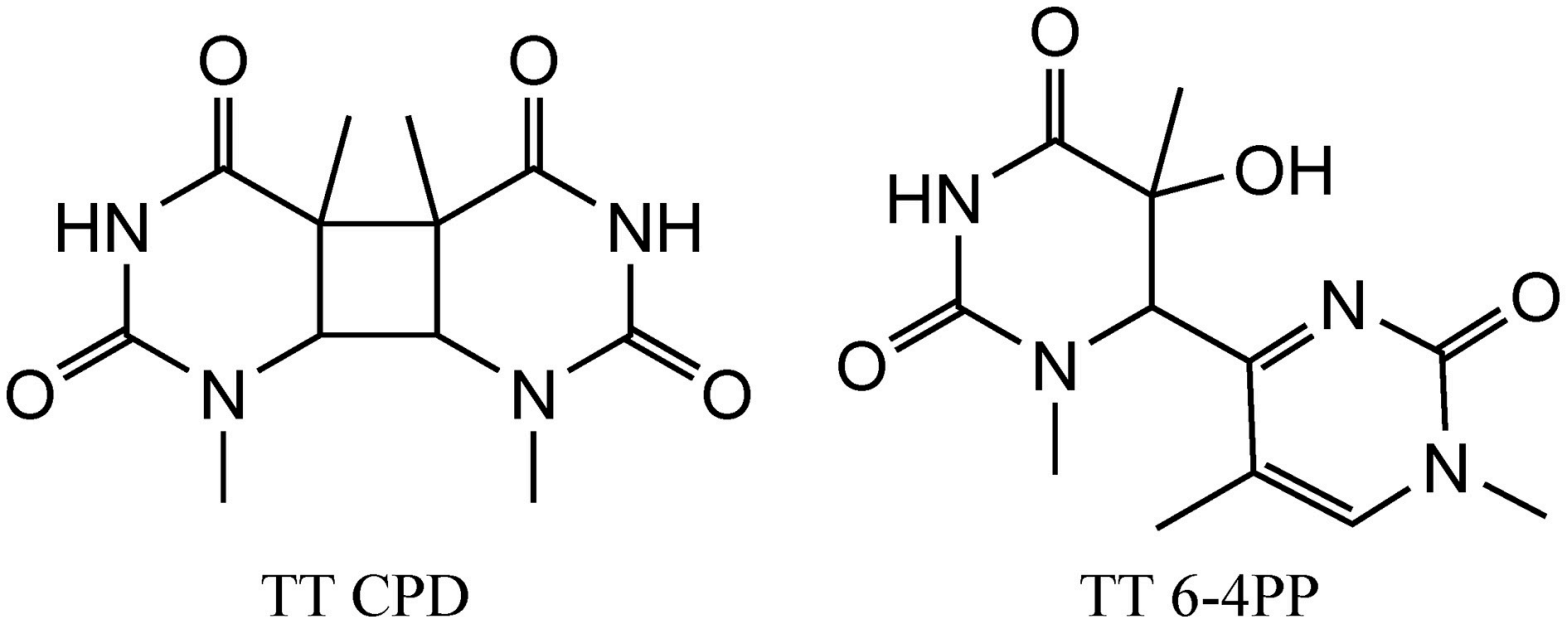
**Structure of the main UV induced pyrimidine dimers, including CPD (left) and 6-4PP (right) dimer generated at TT sequences**.

We initiate our discussion on possible stress DNA damage markers with UV radiation since some of the lesions (as discussed below) induced by this type of radiation are highly specific and characteristic for UV radiation. DNA absorbs UV light mostly in the UVC and UVB ranges and only a little in the UVA wavelengths. However, the UVC light is filtered by the ozone layer of the Earth's atmosphere and does not reach the surface of the Earth. Thus, UVB and UVA represent a major risk factor for the development of skin cancers. Following photon absorption, DNA bases are excited and two major classes of damages can be produced at bi-pyrimidine sequences. Cyclobutane bi-pyrimidine dimers (CPD) are generated through a cycloaddition of the two C5–C6 double bounds of two adjacent pyrimidine bases located on the same strand (Figure 1). The four possible dimeric products, i.e., TT, TC, CT, and CC are produced in cells and skin exposed to UV light (Mouret et al., 2006). The second class of dimeric lesions, identified as a pyrimidine (6-4) pyrimidone photoproducts (6-4PP), are produced by a [2 + 2] cycloaddition between the C5–C6 double bond of the 5′-end base and the C4 carbonyl group of a 3′-end pyrimidine. These above mentioned lesions are specifically produced by UV light and thus could serve as a signature for UV exposure. It has been also shown that UVA can induce the formation of pyrimidine dimers most probably through the excitation of an endogenous photosensitizer that could transfer its energy to DNA bases, generating exclusively CPDs (Mouret et al., 2006). In addition, photoactivated endogenous sensitizers may also induce oxidation of DNA, either directly through a one electron oxidation reaction (Type I) or through the transient formation of singlet oxygen (Type II) (Cadet et al., 2015). These two latter mechanisms are able to produce oxidized DNA bases and mostly 8-oxo-7,8-dihydro-2′-deoxyguanosine (8-oxodGuo) (*vide infra*). This lesion has been extensively used as a biomarker of DNA oxidation, but since it can be produced by several mechanisms, it cannot be indicative of a specific stress. In contrast, pyrimidine dimers (CPD and 6-4PP) are only produced by UV light (Cadet et al., 2000).

### Ionizing radiation and oxidative stress

In cells, following exposure to ionizing radiation, DNA lesions can be produced directly or indirectly. The direct effect induces a one-electron oxidation of DNA, the indirect effect generates ROS through water radiolysis that can subsequently damage DNA. The relative contribution of both effects is still a matter of debates and will be discussed below.

One electron induced DNA oxidation (direct effect) is known to produce mostly guanine damages since this base has the lowest ionization potential among the DNA constituents. Thus, even if oxidation occurs on another DNA base, a fast electron transfer takes place from guanine to the initially produced radical cation that is thus “chemically” repaired (Cadet et al., 2004). Such transfer reaction can take place *in vitro* over large ranges of DNA bases (Hall et al., 1996). The generated guanine radical cation can then decompose through deprotonation or hydration, the second reaction being at the origin of the formation of the well-known 8-oxodGuo.

The indirect effect produces ROS through water radiolysis, among them the hydroxyl radical HO^•^ is the most reactive one, it reacts at a diffusion controlled rate with any biomolecule, including DNA. Reaction of HO^•^ with DNA involves either addition onto aromatic moieties of DNA bases, or through hydrogen abstraction. It has been estimated that about 70% reacts with DNA bases, and 30% with deoxyribose moieties, the latter reaction is giving rise mostly to single strand breaks (SSB). Reaction of HO^•^ with DNA bases involves mostly addition onto aromatic rings giving rise to about 70 different decomposition products. Information on the identified products can be obtained from recent review articles on that topic (Cadet et al., 2012a; Ravanat et al., 2012). It should be highlighted that most of the radiation-induced DNA lesions have been initially characterized using isolated nucleosides or sometimes short oligonucleotides. Thereafter efforts have been made to develop analytical methods (*vide infra*) to search for the formation of these identified modifications first in irradiated DNA following DNA hydrolysis, and when detection sensitivity was high enough, directly in cells exposed to radiation, subsequently to DNA extraction.

**Figure 2:**
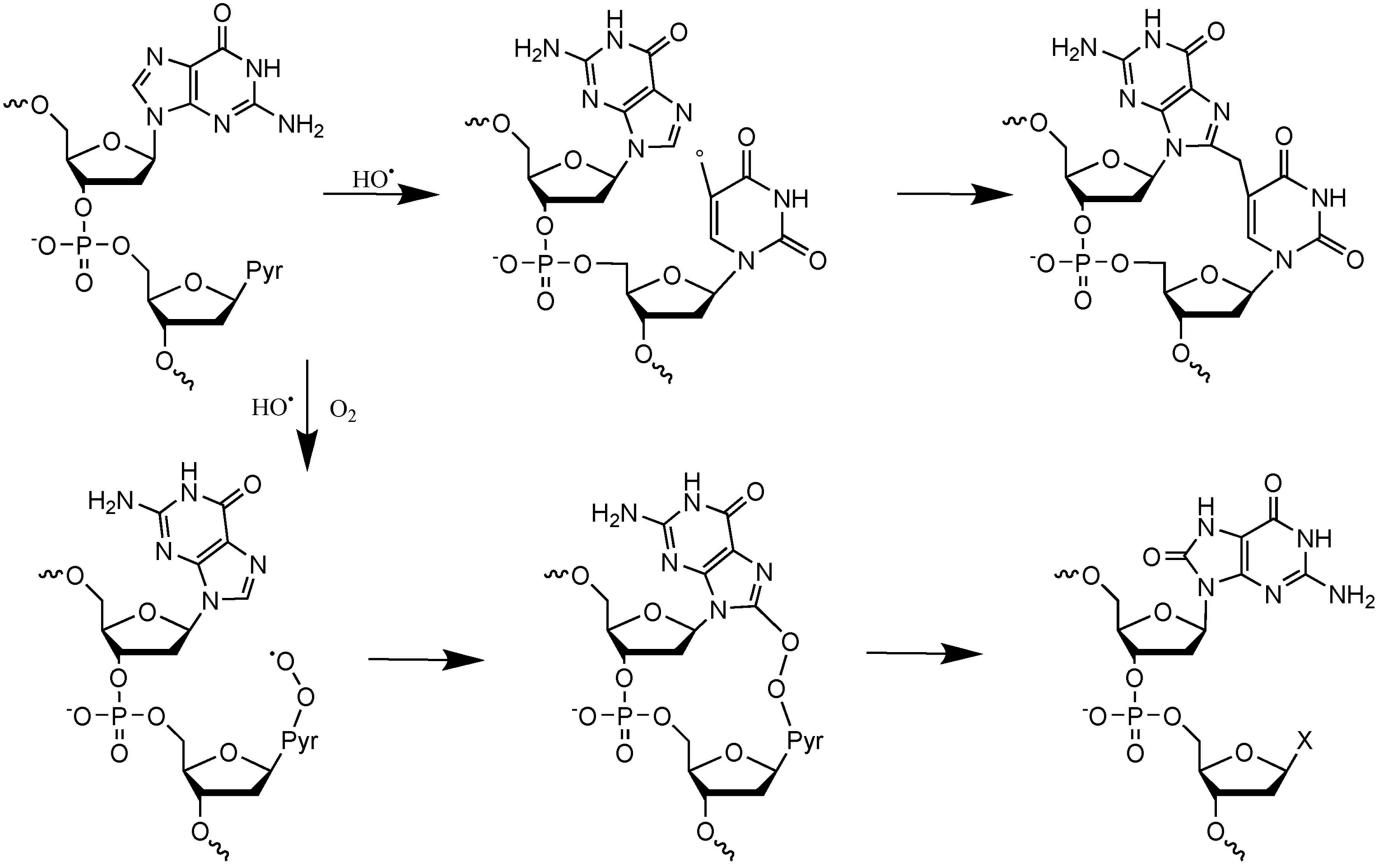
**Mechanisms of formation of tandem DNA lesions induced by HO^•^**. In the absence of oxygen intra-strand crosslinks are produced, whereas in the presence of oxygen tandem lesions containing 8-oxodGuo adjacent to a pyrimidine modification (indicated by an X) are generated.

More recent efforts have been made to study the decomposition reaction of initially produced radicals, directly in dsDNA. A general observation that could be made from the recent data is that initially produced radicals could efficiently react with surrounding DNA constituents to produce complex lesions, including so-called tandem damage involving two adjacent DNA modifications. Two examples of this kind of reaction will be described below in detail; additional information can be obtained from recent review articles (Cadet et al., 2012b; Ravanat et al., 2014). The first one concerns the formation of tandem lesions containing 8-oxodGuo and also 2′-deoxy-7,8-dihydro-2′-deoxyadenosine (8-oxodAdo). These two lesions have been detected in cells exposed to ionizing radiation and one striking observation was that the yield of 8-oxodGuo formation was found to be about one order of magnitude higher than that of 8-oxodAdo (Cadet et al., 2004). It was initially proposed that these two modifications were produced by addition of HO^•^ (indirect effect) onto the C8 atom of guanine and adenine. However, since HO^•^ is very reactive and reacts with a similar efficacy with the two purine bases, such a mechanism, that was supposed to be the predominant one, could not explain the difference in the yield of formation of the two modified purine lesions. More recently, it has been shown *in vitro* that other reactions can generate 8-oxodGuo and 8-oxodAdo. In fact, it has been demonstrated (Bergeron et al., 2010) that the addition of the HO^•^ radical at C8 of guanine and adenine is a minor process (only 5%). Moreover, 50% of the produced 8-oxodGuo has been attributed to the addition of a pyrimidine peroxyl radical onto C8 of an adjacent guanine base, preferably when the purine is located at the 5′ position of the pyrimidine peroxyl radical. Such addition produces an endoperoxide that, following decomposition, gives rise to 8-oxodGuo (or 8-oxodAdo) and an adjacent pyrimidine modification, including formylamine (Bourdat et al., 2000). A third mechanism, that involves an electron transfer reaction, probably from guanine to the initially produced peroxyl radical, explains why formation of 8-oxodGuo is relatively higher than that of 8-oxodAdo. Indeed, as explained previously, such an electron transfer produces mostly guanine damages. Interestingly, in the absence of oxygen, a direct reaction of the pyrimidine radical with a purine base could also occur to generate intra-strand crosslinks (Bellon et al., 2002). These reactions detailed in Figure 2, have been observed *in vitro* using dsDNA, and up to now there is no experimental evidence that implication of the hydroperoxyl radicals is involved in the formation of tandem lesions in cells. However, the fact that at the cellular level the yield of 8-oxodAdo formation is one order of magnitude lower to that of 8-oxodGuo strongly suggests that formation of tandem lesions in cellular DNA following initial formation of a single oxidation event is highly probable. Additional evidence comes from the fact that an unexpected high frequency of spontaneous proximal multiple mutations has been reported in cell and animal models (Hill et al., 2004). Formation of intra-strand crosslinks (produced in the absence of oxygen) has been observed in cells (Hong et al., 2007). It should be also highlighted that the mechanisms of formation of such tandem lesions have been confirmed by theoretical studies (Labet et al., 2008; Dupont et al., 2013).

**Figure 3:**
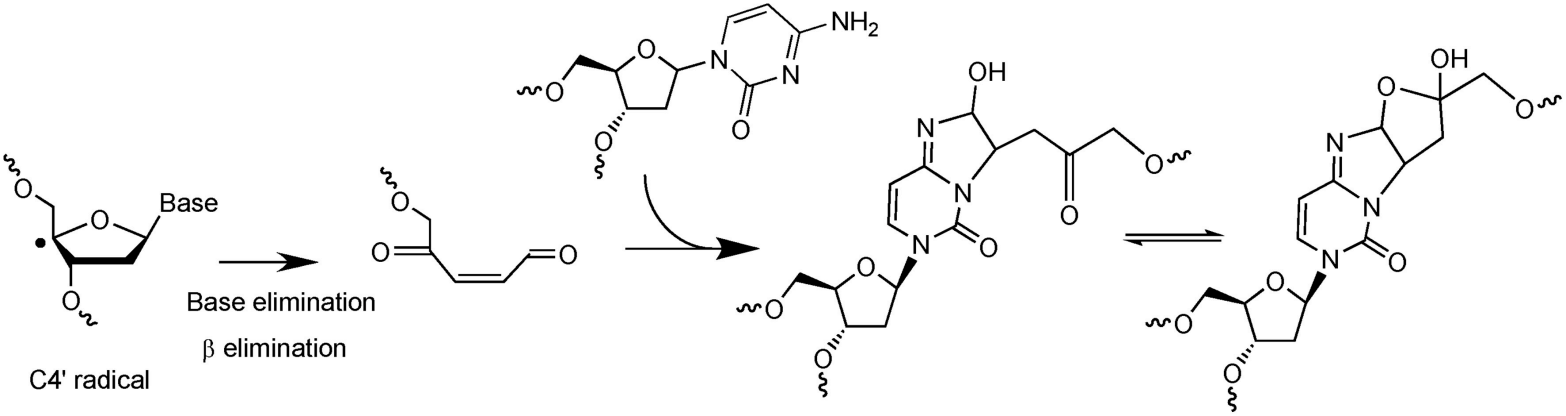
**Mechanism of formation of a complex lesion induced by C4′ hydrogen abstraction**. Following formation of the C4′ radical the produced aldehyde is able to react with a cytosine base located onto the complementary strand to generate an inter-strand crosslink.

The second example of complex DNA lesions produced by a single oxidation event concerns the formation of damages arising from initial 2-deoxyribose oxidation. Such reactions were initially supposed to give rise to SSB when hydrogen abstraction occur on C5′, C4′, or C3′ of 2-deoxyribose. Hydrogen abstraction occurring at C1′ generates an abasic site. These two types of lesions are known to be very rapidly and efficiently repaired in cells. However, it was also reported that reactive aldehydes could be produced following 2-deoxyribose oxidation (Pogozelski and Tullius, 1998). Interestingly, DNA bases are known to react very efficiently with conjugated aldehydes. Using an innovative approach to search for new radiation-induced DNA lesions directly in dsDNA exposed to ionizing radiation (Regulus et al., 2004), a cytosine adduct has been identified and its formation was explained by the initial formation of a reactive aldehyde, as schematized in Figure 3. The first single oxidation event involves hydrogen atom abstraction which occurs at the C4′ position (Regulus et al., 2007). Reaction with oxygen produces a conjugated aldehyde that is then able to react with a surrounding cytosine base mostly located onto the complementary strand (Sczepanski et al., 2008). Thus, the produced lesion implies a strand break and an inter-strand crosslink. This lesion has been also measured in cells, and its yield of formation following exposure to ionizing radiation is within the same range than the formation of DSB. In addition, kinetics of repair of such damage was found to be significantly lower (half-life about 10 h) compared to single lesions that could be totally repaired within a few hours. Other examples of complex DNA lesions produced by initial 2-deoxyribose oxidation have been reported (Dedon, 2008).

**Figure 4:**
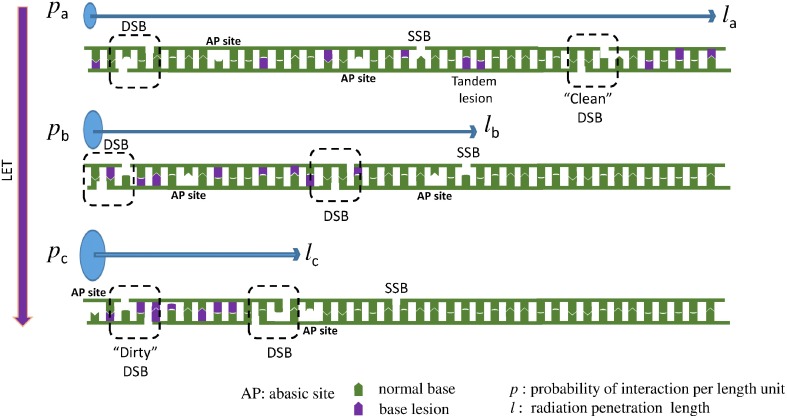
**When LET increases the DNA lesions are denser (clustered)**. Here we present a one dimensional example: Three different particles with the same energy, but with different LET interact with a DNA molecule. Assuming that LET_a_ < LET_b_ < LET_c_, then *p*_a_ < *p*_b_ < *p*_c_, where *p* = P/*l* is the interaction probability per length unit. Thereafter *l_a_* > *l_b_* > *l_c_*, where *l* is the radiation penetration length, the distance that the particle travels until it loses all of its energy inducing different types of DNA lesions, like double strand break (DSB), single strand break (SSB), abasic sites (AP) and oxidized bases. Since all the three particles (low to high LET) carry the same energy and they are able to cause about the same number of lesions and they travel for different distances then the particle with the higher LET will cause more dense lesions, because the same number of lesions (here 2 DSBs, 1 SSB, 8 base lesions, and 2 AP sites) will occur in a smaller distance. This increased complexity is considered a major challenge for the cellular DNA repair systems. To illustrate that increase in complexity, an example of a “clean” DSB containing only two SSB is illustrated for low LET radiation, compared to a “dirty” DSB produced by high LET radiation involving two SSB and base damages. In addition, an example of tandem lesion produced by a single oxidation event is illustrated.

These two above detailed examples of chemical reactions illustrate the complexity of the undergoing reactions taking place when radicals are produced in dsDNA. Since the initial event is the formation of a single radical, these lesions could be produced both by ionizing radiation and also by endogenous oxidative stress. It is important to distinguish these modifications arising from the initial formation of a single oxidation event that we call “tandem lesions” to clustered lesions or so-called multiply damaged sites (MDS) that arose from multiple ionization processes due to the spatial distribution of energy depositions events following exposure to ionizing radiation. However, it is interesting to notice that at the molecular level, up to now, no known specific radiation-induced lesions have been identified, and all identified lesions produced by ionizing radiation could also be produced by endogenous oxidative stress. The main difference is the higher complexity of DNA damage in the case of ionizing radiation as supported by experimental (as reviewed in Hada and Georgakilas, 2008; Georgakilas et al., 2013) and theoretical studies (Nikjoo et al., 2001). This also explains why it is a challenging task to measure radiation-induced DNA lesions, since these lesions are already present in absence of radiation, and their yield of formation per unit dose ~1 lesion per 10 million normal bases and per Gy is relatively low. Thus, the harmful effects of ionizing radiation could not be attributed primarily to the produced single lesions. In fact, more than the chemical nature of the modification, the localization of their formation in clusters could explain the genotoxicity of ionizing radiation (Figure 4). Interestingly, it has been shown that increasing the linear energy transfer (LET) of the particle (Prise et al., 1994) increases the lethality of the irradiated cells due to the high repair resistance of clustered DNA damages (Eccles et al., 2011). Paradoxically, it has been determined that the absolute number of modifications produced per unit dose (Gy), is lower in cells exposed to higher LET particles (such as heavy ions) compared to cells irradiated with gamma or X rays (Pouget et al., 2002). From these observations it could be concluded that the toxicity of ionizing radiation compared to endogenous oxidative stress, is due to the presence of so-called clustered DNA lesions, or MDS (Kryston et al., 2011). In other words, in cells endogenous oxidative stress produces randomly distributed oxidative lesions that could be repaired efficiently by the cell machinery. However, following exposure to radiation, formation of the lesions is localized around the particle track.

When the LET of the particle increases, clusters contain an increasing number of modifications (Tsao et al., 2007; Pachnerova Brabcova et al., 2014). We exemplify this phenomenon in Figure 4. These clustered lesions are difficult to be repair and thus more harmful for the cell. DSB are one of the best examples of these clustered lesions. Their formation through endogenous oxidative stress is very rare since this requires simultaneous oxidation occurring onto the two complementary strands in close vicinity (less than one or two helix turn). With ionizing radiation, the probability of two events taking place locally on the two strands, increases when LET increases. At the chemical level, a damage induced by high LET radiation, will resemble at a mixture of the above 70 different identified DNA lesions plus a SSB (Stewart et al., 2011; Georgakilas et al., 2013). This illustrates the complexity at the molecular level of the lesions that could be produced in the DNA of cells exposed to ionizing radiation. Only little information is available regarding the formation of radiation-induced DNA-protein crosslinks. At the molecular level, only a few examples of reactions involving amino acids and DNA bases have been reported. Formation of guanine-lysine adducts generated through a one-electron oxidation reaction following nucleophilic addition of ε-amino group of lysine onto C8 of guanine is one of the possible mechanisms (Perrier et al., 2006). Further work has to be done to better estimate the importance of such damages in cells (Jaruga and Dizdaroglu, 2008).

## Methods for measuring DNA lesions

Measuring DNA damage in cells is a challenging analytical problem since the level of damages to be measured is relatively low and represents generally less than one modification per million normal bases. In addition, such a measurement has to be performed with a limited amount of biological material. Two different strategies have been used for this purpose.

Direct methods are based on analytical chemistry, requiring first extraction and then digestion of DNA, followed by the measurement of specific DNA lesions (at the nucleotide, nucleoside or base level) using a more or less specific detector coupled to a chromatographic separation.

Indirect biochemical methods have been also developed. These assays measure generally strand breaks. By coupling to DNA repair enzymes that convert lesions into strand breaks, or by using specific antibodies raised against DNA lesions, several modifications can be quantified. More recently, antibodies were raised against protein or protein modifications belonging to the DDR mechanisms allowing detection of “repair” foci of active repair that could be directly correlated to DNA damages. Finally, biological consequences of the generated lesions, such as micronuclei or chomosomal aberrations could be also used to monitor damages to DNA.

### Direct methods

The principle of the direct measurement of DNA lesions involves first extraction of genomic DNA from the cells, followed by the complete hydrolysis to monomeric units, being either nucleotides, nucleosides or bases (Ravanat, 2012). Then analytical methods are used to separate the hydrolyzed products and a specific detection method is used to detect and quantify the lesions.

High performance liquid chromatography coupled to electrochemical detection (HPLC-ECD) was one of the first methods developed in the early eighties for the detection of 8-oxodGuo (Floyd et al., 1984). As this lesion has an ionization potential lower than that of normal bases, it can be detected selectively and quantitatively. This approach has been also extended to a few other DNA modifications including 5-hydroxy-2′-deoxycytidine (5-HO-dCyd) and 8-oxodAdo. The system is also usually equipped with a less sensitive UV detector that monitors normal bases, and thus results could directly be expressed as the number of modification per million normal nucleosides, in enzymatically hydrolyzed DNA samples.

During the same period of time, a method based on gas chromatography coupled to mass spectrometry (GC-MS) has been also developed (Dizdaroglu, 1984). Such a method has been applied to acid hydrolyzed DNA samples that release free bases. This method requires also an additional derivatization step since DNA bases are not volatile enough to be separated by GC. The derivatized products could then be detected by mass spectrometry in the so-called selected ion monitoring (SIM) mode. This approach is thus more versatile than the HPLC-ECD method that is limited to the detection of only a few DNA lesions. However, it has been rapidly highlighted that the two methods reported significant different background levels of lesions measured in eukaryotic cells in the absence of any stress (Halliwell and Dizdaroglu, 1992). At the end of the last century, it was clearly established that the difference was attributed to the derivatization step of the GC-MS assay that, by oxidizing DNA bases (Ravanat et al., 1995), artefactually creates DNA lesions and thus provides overestimated levels of damages (Cadet et al., 1997).

Nowadays, HPLC coupled to tandem mass spectrometry (through electrospray ionization, HPLC-MS/MS) is the method of choice for measuring low levels of DNA damages (Ravanat, 2012). This method, due to a soft ionization technique, is very sensitive, at least in the so-called multiple reaction monitoring (MRM) mode that requires tandem mass spectrometry. Isotopically labeled internal standards could be used to increase the accuracy of the quantitation. If an analytical tool is currently available, it should be kept in mind that artefactual formation of DNA lesions during the work-up, including DNA extraction, is always possible. Thus, through a European collaborative project named “European Standard Committee on Oxidative DNA Damage” (ESCODD) (ESCODD, 2002, 2003) efforts have been made to minimize spurious oxidation that could occur during the work-up. Optimized protocols are now available (Ravanat et al., 2002).

Using HPLC-ECD and HPLC-MS/MS (and appropriated protocols for DNA extraction and hydrolysis) the amount of a several DNA lesions has been measured in cells exposed to increasing doses of gamma irradiation (Pouget et al., 1999, 2002). Thymidine glycols were found to be the major lesions and their yield of formation, around 0.1 modification per 10^6^ bases and per Gy, was found to be 4 times higher than that of 8-oxodGuo, whereas a recent work reported similar levels for the two lesions (Madugundu et al., 2014). Interestingly, the yield of formation of the formamido-pyrimidine derivative of guanine was higher (about 2 times) to that of 8-oxodGuo suggesting that the DNA is in a reducing cellular environment since both lesions arose from the same intermediate and formation of 8-oxodGuo requires oxidizing conditions. It should be noticed that the complex lesion involving formation of cytosine adducts was also detected in cells by HPLC-MS/MS and its level of formation is about two orders of magnitude lower than that of 8-oxodGuo (Regulus et al., 2007), and thus almost similar to that of DSB estimated to be about 40 DSB per cell and per Gy. More recently 2′,3′-dideoxyribonucleosides were also measured in cells exposed to ionizing radiation (Madugundu et al., 2012). Their yield of formation is relatively low, similar to that of DSB, but they are supposed to be specifically generated by secondary electrons with an average kinetic energy of 10 eV and they can react with DNA components by a mechanism involving dissociative electron attachment (Sanche, 2009; Park et al., 2013; Kouass Sahbani et al., 2014). Further work has to be done to clearly establish the role of these low energy electrons to induce strand breaks and base damage at the cellular level.

### Indirect methods

Indirect or biochemical approaches have been also used to detect DNA lesions in cells. First attempts have been made to use antibodies raised against DNA lesions. Thus, specific antibodies raised against pyrimidine dimers were developed and were found to be specific enough to detect formation of these UV-induced lesions (Perdiz et al., 2000). For oxidative DNA lesions, and mostly 8-oxodGuo, the developed antibodies were found to be not specific enough and thus cross-reaction with guanine base was found to give overestimated results (Breton et al., 2003).

Other indirect methods are based on the detection of DNA strand breaks. Among them, the alkaline elution (AE) or the more recently developed comet assay are well suited. These methods enable the measurement of strand breaks (mostly single strand breaks: SSB) based on the fact that an alkaline elution of DNA through a filter is faster if it contains breaks (AE) (Pflaum and Epe, 2000), or that electrophoresis of DNA embedded in an agarose gel is increased in the presence of a SSB (single cell gel electrophoresis—SCGE or “Comet” assay) (Boysen et al., 2010). To increase the versatility of the assay, the approach could be combined with DNA repair enzymes like human (OGG1, NTH1) or bacterial glycosylases (Fpg, EndoIII) that excise oxidative DNA lesions and thus induce additional breaks. Thus, prior to electrophoresis (or elution), DNA can be treated by these glycosylases and thus the additional strand breaks are interpreted as the base modifications that have been recognized by the DNA repair enzymes. By running the electrophoresis under alkaline (denaturing) conditions, total lesions are measured, while under neutral (non-denaturing) conditions, bistranded DNA lesions, i.e., two lesions located on opposing strands, are quantified. An adaptation of this approach for the detection of bistranded clustered DNA lesions is presented in the next section (Georgakilas et al., 2010; Georgakilas, 2011). Coming back to the idea of using DNA repair enzymes as damage probes, for example, it is accepted that Fpg-sensitive sites are mostly due to the presence of oxidized purine bases, including mostly 8-oxodGuo. Using such an approach the relative proportion of direct strand breaks (including also alkali-labile sites), oxidized purine and pyrimidine bases has been determined in cells exposed to ionizing radiation (Cadet et al., 1999). Formation of these lesions was found to increase linearly with the radiation dose (0–20 Gy). The amount of Fpg-sensitive sites was found to be similar to that of EndoIII sensitive sites, suggesting that an almost similar amount of oxidized pyrimidine and purine bases is produced. In addition, the number of direct strand breaks (including also alkali-labile sites) was found to be similar to the number of modified bases. As already mentioned, increasing the LET of the particle was found to lower the yield of formation of the individual lesions (Pouget et al., 2002). For comparison it has been demonstrated that singlet oxygen only produced 8-oxodGuo in cellular DNA (Ravanat et al., 2000), in the absence of significant formation of strand breaks (Ravanat et al., 2004).

Using the specific measurement of several DNA lesions, attempts have been made to determine the relative importance of the direct vs. indirect effect (Douki et al., 2006). This remains to determine the relative proportion of lesions produced by a one-electron oxidation mechanism, compared to lesions produced by HO^•^. As mentioned above, one-electron oxidation of DNA produces mostly 8-oxodGuo, and this has been demonstrated experimentally using a two photons ionization system (high intensity 266 nm laser pulses) (Douki et al., 2004). HO^•^ produces several lesions, including also 8-oxodGuo. Thus, one would expect that increasing the LET of the radiation, that is supposed to increase the proportion of the direct effect, would also increase the relative formation of 8-oxodGuo. However, this was not observed experimentally, strongly suggesting that the direct effect plays a minor role in the formation of the radiation-induced DNA lesions. Additional experiments are required, using lesions specifically produced by an one electron oxidation reaction (that is not the case for 8-oxodGuo that could be also produced by hydroxyl radicals) to confirm such results. Recently, identified polyamine-guanine adducts (Silerme et al., 2014) could potentially be used for such a purpose.

### Others methods to measure DNA damages through their consequences

Another possible approach to measure cell damage is flow cytometry. Since flow cytometry gives information of cell size and fluorescence intensity, the most explored application in the frame of DNA damage is the detection of aneuploidy or polyploidy. More precisely, speaking for oxidatively-induced DNA damage, the application of flow cytometry is to explore the relative levels of fluorescence between treated and untreated cells, stained with antibodies binding to oxidative stress related proteins. This is a rather old but relatively rapid and reliable technique, while it is quite indirect, since it measures changes in light scattering and fluorescence of nucleoids after cellular irradiation (Milner et al., 1987). Newer approaches target simultaneously DDR proteins (by use of specific antibodies) as a marker of DNA damage like DNA repair proteins γ-H2AX and BER enzymes. For example Ong et al. report the first estimation of OGG1 levels by flow cytometry (Peng et al., 2003). The most explored DDR proteins using flow cytometry are TP53 (Sarasqueta et al., 2013), γ-H2AX (Li et al., 2013), CHEK1, ATR, ATM, TP53BP1, CASP3, and PRKDC.

In addition, an alternative way to measure DNA lesions is to measure the consequences of the produced damages. This concerns for example the measurement of the mutations induced by the damages or chromosomal abnormalities, using for example the micronuclei test or determining chromosomal aberrations. These methods will not be described in the present article.

### Measuring clustered DNA lesions (DSBs and non-DSB lesions)

There is a quite limited number of methodologies for detecting and measuring clustered DNA lesions and especially non-DSB lesions. An alternative approach to measure DNA lesions is to measure the activation of the DDR system that is triggered following formation of lesions. This approach has been extensively used to assess in cells the formation and repair of DSB. Indeed, following formation of a DSB, the ATM protein is able to phosphorylate a histone variant H2AX located nearby the DSB, the purpose of such phosphorylation is to signal to the DNA repair machinery the presence of a damage. Antibodies raised against the phosphorylated form of H2AX, named γ-H2AX, allow to detect foci of the phosphorylated protein that could then been attributed to the presence of a DSB (Rothkamm and Horn, 2009). These foci could be directly observed using a fluorescent microscope and counted to determine the number of DSBs. Measurement at different time points after irradiation allows determination of the repair kinetics of radiation-induced DSBs. Other proteins (like MRE11, 53BP1 etc.) involved in DNA repair could be used in a similar way to localize and follow over time the presence and processing of radiation-induced DNA lesions. The sensitivity of γ-H2AX immunofluorescence is high, with one focus corresponding to one DSB, and ca. 20–30 foci induced per Gy and per cell. Therefore, all these methodologies can be applied to relative low doses of a few mGy up to 2–3 Gy where usually saturation is reached.

### Electrophoretic approaches

The most reliable quantitative approaches to measure DSB are considered to be the electrophoretic ones like Pulse Field Gel Electrophoresis (PFGE) and its various adaptations using DNA repair enzymes as described above. First experimental evidence for the existence of clustered DNA damage following exposure to low- or high-LET radiation was provided in the 90's as described in these reviews (Hada and Georgakilas, 2008; Georgakilas et al., 2013). One major breakthrough though has been done by Sutherland et al. measuring very accurately different types of clustered DNA lesions using a sensitive adaptation of non-denaturing electrophoresis (Sutherland et al., 2000). Focusing on the bistranded DNA lesions i.e., DSBs and non-DSB oxidative clustered DNA lesions (OCDLs) (Georgakilas, 2011), we rely on the fact that repair enzymes participating in BER like DNA glycosylases and AP endonucleases will function also *in vitro*, i.e., on isolated DNA carrying different patterns of non-DSB lesions. Once they detect the lesion in each strand and in each cluster, they will excise it and cleave the DNA strand 3′-prime to the DNA lesion by their intrinsic lyase activity (DNA glycosylases: human OGG1 or NTH1) or cleave directly 5′-prime to the abasic (AP) site in the case of an AP endonuclease, like human APE1 and create a SSB in each strand, i.e., a DSB in the case of a cluster. These additional indirect DSBs which are formed *in vitro* by the assay are different from the ones induced directly by the irradiation. Therefore, in the same gel both DSBs and OCDLs can be measured using any electrophoresis combined with number average length analysis (NALA) (Sutherland et al., 2003). The sensitivity of the assay is quite satisfactory and while for DSBs there is a limiting dose of 3–5 Gy where one can measure reliably these lesions, for OCDLs the dose can be even below 1 Gy with the use of DNA repair enzymes and appropriate electrophoretic protocols (Sutherland et al., 2002). By the use of the above approaches one can measure a few DSBs and OCDLs per Gy per cell and the suggesting ratio is 1 DSB: 3-5 OCDLs for a wide range of doses (Hada and Georgakilas, 2008; Georgakilas et al., 2013). A significant methodological improvement for measuring DSBs and OCDLs in individual cells has been done by different groups using different adaptations of the Comet assay (Blaisdell and Wallace, 2001; Georgakilas et al., 2010). The adaptations of the Comet assay under alkaline (denaturing) conditions offer a high sensitivity and detection of DNA damage even in the dose range of a few mGy up to 1–2 Gy. The drawback in this case is the fact that this assay is not as quantitative as the PFGE, and thus does not allow quantifying the number of lesions per cell per Gy. Another possible disadvantage of all electrophoretic approaches is that the running conditions and the so-called “electrophoretic regime” have to be carefully chosen, so the small DNA fragments not to be “lost” during the electrophoresis and therefore DSBs and OCDLs to be underestimated (Hada and Georgakilas, 2008). The importance of small DNA fragments usually less than 10 kbp becomes very important in the case of high-LET radiation where the complexity of DNA damage and therefore the proximity of DNA lesions are expected to be high and in general much higher than that of low-LET radiations.

### Immunostaining

For the detection of DSBs and OCDLs using other more modern methods, “DNA staining” methods were adapted lately, such as immunohistochemistry (IHC), immunocytochemistry (ICC), immunofluorescence (IF), flow cytometry, ELISA and Western Blotting. These techniques utilize properly developed antibodies in order to detect the presence of a specific protein. A general description of an antibody could be like a Y-shaped protein, that its upper region, the edges of the branches, is devoted to the recognition (recognition area) of the so-called antigen, while the trunk of the Y is the functional region. Immunofluorescence is the branch of immunostaining that uses fluorescence dyes for the visualization of the target molecules in the fluorescence microscope.

Among DNA lesions, the most hazardous is accepted to be the DSB, since this lesion may lead to changes of DNA sequence and thereby into mutations if repaired by the error-prone NHEJ and not through HR pathway. As discussed above, a very informative DSB repair protein is the H2AX, which under a DSB turns into its phosphorylated form (γ−H2AX). γ-H2AX forms foci that can be microscopically observed as fluorescent spots after immunofluorescence staining. The γ−H2AX assay is considered as the gold standard of DSB detection, since a linear dependence of foci number on radiation dose has been verified and there is a “1:1” correlation between foci number and DSBs. Since the importance of γ−H2AX is given, it is a worthwhile task to look for sites of colocalization of γ-H2AX foci with potent foci of other DDR proteins. Both epifluorescence and confocal microscopy can be utilized. Although confocal is by definition more informative, epi-fluorescence could be approved as more time-effective. γ−H2AX foci detection can be carried out through epifluorescence using a high throughput image analysis software, making foci imaging and scoring almost fully automated (like the Metasystems Metafer 4 system).

**Figure 5:**
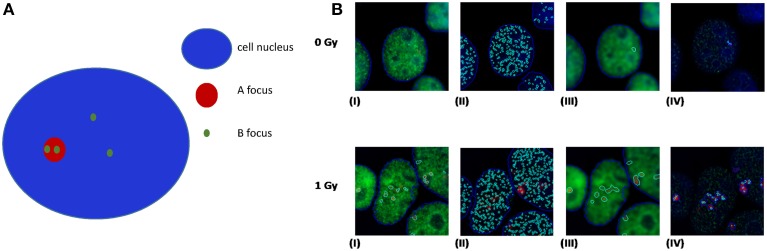
**(A)** Principles of colocalization between foci of dissimilar size. Colocalization exists if the ratio of B foci number per A nucleus area is greater than the ratio of B foci number per cell nucleus area. **(B)** Realistic example of colocalization between APE1 and γ-H2AX foci in human HepG2 cells irradiated with high-LET Ar heavy ions (Ar-36, LET 269.4 keV/μm). In our case γ-H2AX forms large and bright red foci with clear boundaries, while APE1 gives a punctuated and diffused staining, forming numerous small “foci,” even in case of non-irradiated cells. In an attempt of using a freely available software, we have performed the analysis using the Jcount software (courtesy of Dr. Pavel Lobachevsky group, Peter McCallum Institute, Australia). As expected a significant increase in the colocalization of APE1 foci with the DSB focus is observed for all irradiated samples compared to non-irradiated (0 Gy). (I)–(IV): 0 Gy 1 h and 1 Gy 1 h. (I) γ-H2AX foci (per cell); (II) APE1 foci (per cell); (III) complex γ-H2AX foci that would serve as the area for estimation APE1 foci per complex γ-H2AX focus in (IV).

A recent publication by Asaithamby et al. (2011) utilized the idea of detecting clustered DNA lesions in human cells using the foci colocalization approach for low and high-LET ions. Generally speaking, colocalization is referred to the detection of two different color foci (each one corresponding to a different DDR protein) that coexist in the same cell area. For calculating the level of co-localization, highly specific software like the Imaris (Bitplane) can be used. Usually the criterion for foci colocalization is the coverage of a minimum percentage (e.g., 70%) of focus A area by the focus of protein B and vice versa. This is useful for estimation of co-occurrence between foci of similar size. As an example and using a freely available software (JCount), colocalization of APE1 and γ-H2AX foci in HepG2 cells irradiated with argon ions (Ar-36, LET 269.4keV/μm) is presented (Figure 5). In this case one can see that γ-H2AX forms large and bright foci with clear boundaries, while APE1 gives a punctuate and diffused staining, forming numerous small “foci,” even in case of non-irradiated cells.

The origin of this difference is that γ-H2AX appears only after induction of a DSB, while APE1 protein pre-exists in cells, and under a DSB, it is just localized at the site of damage. Moreover, one γ-H2AX focus may extend through 2 × 10^6^ bp, consisting of about 2000 molecules of phosphorylated histone H2AX (Rogakou et al., 1998), while in case of APE1 the task is to identify the migration of some tens of molecules at the site of DSB. There are however some limitations in this approach. It cannot be easily claimed that one is able to detect the fluorescence of individual molecules and thus to count the number of APE1 or other repair protein molecules (corresponding to abasic sites or oxidized bases using for example OGG1), thereby specifying precisely the number of non-DSB lesions that accompany a DSB. Taking into account these limitations, it is a logical consequence that the resulting non-DSB foci number is highly dependent on software specified parameters. In addition and toward the optimization of this methodology, the use of high-LET particles might be helpful since the colocalization can be tested on the particle track and not anywhere in the cell like in the case of X- or γ-rays. Last but not least and according to our experience, the first step for unbiased foci analysis is to capture comparable images. For that the microscope imaging software can be set to integrate for different but specified intervals for each fluorochrome. The proper integration time, i.e., the period of time that the CCD camera collects the emitted fluorescent light, can be defined as the maximum that for the highest signal to observe no color oversaturation. By this way, a reliable evaluation of colocalization between two proteins can be performed like for example APE1 and γ-H2AX.

## Future developments

### Utilizing meta-analysis tools in order to reveal potent and existent DNA damage biomarkers

Toward the necessity of developing reliable markers for different types of stresses inducing DNA damage and under consideration of the overlap between the different DDR pathways including DNA repair, we use meta-analysis tools, wishing to suggest possible biomarkers for future applications.

Based exclusively in previously published data, we present in this section suggestions for potent DNA damage biomarkers. Our results are presented in two tables. Considering that the three more hazardous types of DNA damage are due to: (a) exposure to ionizing radiation, (b) occurrence of oxidative stress, and (c) replication stress, in Table 1, we suggest that the products of those DDR and repair genes could serve potentially for “exclusive” identification of each stress type. Thus, the usage of the DDR genes of Table 1 targets the detection of the type of the stress occurring in the cell through the type of DDR response initiated. In Table 2, we quote the most common genes that have been correlated in literature with the above mentioned three situations. We must mention that the genes in Table 2 can participate in more than one DDR pathways or types of responses in contrast to Table 1, that each gene can be assigned with a relative safety to a specific type of stress-induced DNA damage, for example radiation-induced DDR. Thus, the usage of Table 2 is to suggest in general genes for the confirmation and quantitative assessment of existent DNA damage not knowing necessarily the type of agent or situation which induced it. In order to mine the genes for these two tables, two independent sequences of searching were performed.

**Table 1:** **DNA repair genes that could serve for better identification of exposure to ionizing radiation, occurrence of oxidative stress or identification of DNA replication processes**.

**Response to ionizing radiation**	**Response to oxidative stress**	**DNA replication**
*FAM175A*	*PSEN1*	*POLE3*
*BRE*		*RECQL5*
*BABAM1*		*IGHMBP2*
*UIMC1*		*POLG2*
*NABP2*		*RFC2_HUMAN*
*NABP1*		*RFC4_HUMAN*
*EYA3*		*RFC3_HUMAN*
*EYA1*		*RFC5_HUMAN*
*BRCC3*		*RFA1_HUMAN*
*INIP*		*RFA3_HUMAN*
*INTS3*		*RFA2_HUMAN*
*RNF8*		*INO80E*
*RNF168*		*RNASEH2A*
*C10orf90*		*ATR_HUMAN*
*RFWD3*		*RBM14*
*AEN*		*CDK2_HUMAN*
*USP28*		
*BRSK1*		
*PAXIP1*		

*The analysis was based only in literature available data running multiple times the GLAD4U, testing specific query terms as described in the text. For the evaluation of the results, some genes and terms were further sampled in other search engines like Quertle*.

**Table 2:** **Genes most commonly used in bibliography in order to identify exposure to ionizing radiation, occurrence of oxidative stress and DNA replication process**.

**Ionizing radiation**						**Oxidative stress**		**Replication stress**	
**Score**	**gene**	**score**	**gene**	**score**	**gene**	**Score**	**gene**	**Score**	**gene**
194.8	*ATM*	31.8	*ERCC5*	15.5	*RAD9A*	127.8	*OGG1*	113.6	*ATR*
87.5	*DDB2*	29.9	*XRCC6*	14.5	*FANCD2*	43.7	*APEX1*	48.3	*CHEK1*
79.3	*TP53*	29.9	*TP53BP1*	14.4	*RNF8*	29.2	*SOD2*	42.5	*ATM*
73.5	*H2AFX*	29.4	*ERCC2*	14.2	*RAD51B*	28.1	*PARP1*	32.4	*CLSPN*
71.7	*NBN*	29.0	*XRCC3*	14.2	*CDKN1A*	27.3	*ATM*	29.0	*ATRIP*
71.2	*PRKDC*	29.0	*XRCC5*	13.8	*BARD1*	23.7	*GSTM1*	23.8	*RPA1*
56.5	*ATR*	27.3	*XRCC4*	13.6	*NTHL1*	23.3	*XRCC1*	20.8	*RPA2*
55.2	*CHEK2*	23.5	*APEX1*	13.6	*CDC25C*	18.7	*CAT*	17.0	*H2AFX*
54.6	*MRE11A*	22.8	*DCLRE1C*	13.4	*XRCC2*	18.5	*GSTT1*	15.6	*SMARCAL1*
53.8	*BRCA1*	22.1	*RAD18*	13.3	*NHEJ1*	17.9	*GPX1*	14.3	*BLM*
52.8	*XRCC1*	21.1	*OGG1*	13.3	*BRCA2*	17.0	*ERCC6*	14.0	*RAD17*
52.7	*CHEK1*	19.5	*CDC25A*	13.0	*ATRIP*	16.8	*NFE2L2*	13.7	*WRN*
52.3	*RAD51*	19.5	*RAD52*	12.8	*ERCC1*	15.7	*ERCC2*	11.4	*CDC25A*
51.1	*XPA*	19.1	*ERCC8*	12.5	*LIG4*	13.9	*NUDT1*	10.5	*RAD9A*
50.3	*DDB1*	18.4	*ERCC4*	12.0	*POLD1*	12.5	*NEIL1*	10.3	*FANCM*
47.1	*PCNA*	18.2	*CUL4A*	11.8	*PNKP*	11.4	*WRN*	10.3	*NBN*
44.9	*XPC*	17.9	*ERCC3*	11.5	*KAT5*	11.0	*MUTYH*		
39.8	*ERCC6*	16.7	*UIMC1*	11.4	*MDM2*	10.8	*TP53*		
39.6	*RAD50*	16.5	*RPA1*	10.4	*PPM1D*				
37.6	*MDC1*	16.4	*PARP1*	10.2	*ABL1*				
32.2	*POLH*	16.0	*RPA2*						

*In this table and in contrast with Table 1, these proteins are not suitable for exclusive identification, but there are the most informative, although some of proteins seem to appear only in one category. That is because in Table 2 only the proteins with the higher score are displayed. In each case, the term “score” as it arises in GLAD4U. “Score” is a measure of relevance between the query terms, it serves for the prioritization of the results. It is defined as the negative logarithm of the hypergeometric p-value*.

Firstly, we run multiple times the *GLAD4U* (Jourquin et al., 2012), testing different query terms. In order to define the most suitable genes for oxidative stress we unified the results of the query terms: “oxidative stress and DNA repair,” “oxidative stress and DNA Repair,” “DNA repair and oxidative stress.” We kept only those with score greater than 10, as it arises in *GLAD4U*. Score is a measure of relevance between the genes and the query terms, it serves for the prioritization of the results. It is defined as the negative logarithm of the hypergeometric *p*-value. Respectively, the same was done for the other two entries. “Ionizing radiation and DNA repair,” “DNA repair and ionizing radiation,” and “ionizing radiation and DDR” were unified in order to produce the gene list for “Ionizing Radiation.” Accordingly, for “Replication Stress” the results of the queries “DNA repair and replication stress,” “replication stress and DNA repair” and “replication stress and DDR” were taken into account. Especially for replication stress PANTHER library was also utilized. For the evaluation of the results, some genes and terms were sampled in other search engines (like Quertle, LLC, 2014). Thus, Table 2 was created. We have to emphasize here, that genes of Table 2, in contrast with Table 1, are not suitable for “exclusive” identification of the nature of the DNA stress, although some of them seem to appear only in one category. This happened because we kept only those genes with score greater than 10.

For Table 1 in addition to the previous analysis, a second one was performed. We searched with *AmiGO2*, a tool of the *Gene Ontology Consortium* search engine (The Gene Ontology, 2015), using the proper of the available filters. The resulting genes were tested with *BioVenn* (Hulsen et al., 2008) in all combinations with the other two categories, and also with the categories that resulted with *GLAD4U*. We kept only the unique genes of each category. To enhance the significance of our results, we run extra tests using other search engines (see below). Our results indicate a variety of gene products that maybe possibly used as “signatures” for each type of stress and be further tested experimentally. We cannot exclude the existence of other possible genes that equally or better characterize for example exclusively radiation response and not replication nor oxidative stress. Our findings were based on published data and the specific search engine tools (see supporting information).

### Evaluation of meta-analysis results by manual searching in quertle and other bibliographic data bases

In order to more efficiently scrutinize our results we performed for all of the above found genes (Table 1) a manual search in Quertle (LLC, 2014) and also using databases GeneCards (Safran et al., 2010), Uniprot (Apweiler et al., 2004), Entrez Gene (Maglott et al., 2005) and PANTHER (Moussavi Nik et al., 2012). No Quertle search result overlap was found for any of these genes between each of them and the other two categories (columns). For example, ionizing radiation genes (column 1) with the terms “oxidative or replication” stress suggesting a possible “uniqueness” for each of these markers. As a result, these genes or their encoded proteins are potential biomarkers, but they need to be validated in further studies. Some examples are listed below:

### Ionizing radiation

- ***FAM175A, BRE, BRCC3, BABAM1, and UIMIC1***: They are all located in different chromosomes and their protein products belong to the BRCA1-A complex that is involved in DDR and DSB repair. The BRCA1-A complex specifically recognizes “Lys-63”-linked ubiquitinated histones H2A and H2AX at DNA lesion sites, leading to target the BRCA1-BARD1 heterodimer to sites of DNA damage at DSBs. This complex also possesses deubiquitinase activity that specifically removes “Lys-63”-linked ubiquitin on histones H2A and H2AX+ (Apweiler et al., 2004). This complex takes part in G2 DNA damage check point and particularly participates in X-ray induced DDR.
- ***NABP1 and NABP2***: They are components of the SOSS complex, a multiprotein complex that functions downstream of the MRN complex to promote DNA repair and G2/M checkpoint. In the SOSS complex, NABP1 and NABP2 act as a sensor of single-stranded DNA that binds to single-stranded DNA, in particular to polypyrimidines. The SOSS complex associates with DNA lesions and influences diverse endpoints in the cellular DDR including cell-cycle checkpoint activation, recombinational repair and maintenance of genomic stability. Required for efficient HR-dependent repair of DSBs and ATM-dependent signaling pathways (Safran et al., 2010).

### Oxidative stress

- ***PSEN1***: It was rather surprising for us that only one gene “survived” our screening as described above. The close bonds between oxidative stress and ionizing radiation when it comes to induction of DNA damage can probably explain this result i.e., very difficult to identify oxidative-stress unique genes. According to Gene Ontology, PSEN1 is a gene belonging in the DDR family of genes and especially damage resulting from oxidative stress inducers like hypoxia (Moussavi Nik et al., 2012). The encoded protein belongs to gamma secretase complex, which is a cellular component. Gamma-secretase cleaves several transmembrane proteins including the cell surface receptor Notch and the beta-amyloid precursor protein. Presenilin is a protein which forms a complex with Aph1, Nicastrin and others to cause intramembranous proteolysis of Notch subsequent to its extracellular cleavage by TACE after ligand binding. Presenilin is the actual peptidase in the complex. Its name derives from the fact that it is separately also the peptidase involved in cleavage of the β-amyloid precursor protein or β-APP, and in this role, mutations in Presenilin1 and Presenilin 2 in humans have been linked to familial early onset Alzheimer's disease as researched in PANTHER database database (Mi et al., 2005). It also participates in metabolic processes, through oxidoreductase activity. PSEN1 interacts selectively and non-covalently with oxygen. Searching in Quertle that provides correlated results, gave no direct results when we combined this gene with ionizing radiation or replication stress. Collectively, PSEN1 has been correlated with signal transduction in response to DNA damage, and activation of MAPKK activity.

### DNA replication

- ***POLE3***: is a histone-fold protein that interacts with other histone-fold proteins to bind DNA in a sequence-independent manner. These histone-fold protein dimers combine within larger enzymatic complexes for DNA transcription, replication, and packaging (Maglott et al., 2005).
- ***RECQL5***: is a DNA repair gene, the encoded proteins of which contribute to DNA replication being responsible for DNA duplex unwinding. It is required for mitotic chromosome separation after cross-over events and cell cycle progress. It is required for efficient DNA repair, including repair of inter-strand cross-links. Stimulates DNA decatenation mediated by TOP2A. It prevents sister chromatid exchange and HR (Apweiler et al., 2004).
- ***IGHMBP2***: This gene encodes a helicase superfamily member that binds a specific DNA sequence from the immunoglobulin μ chain switch region (Maglott et al., 2005). The encoded protein is a 5′–3′ helicase that unwinds RNA and DNA duplexes in an ATP-dependent reaction. It acts as a transcription regulator and is required for the transcriptional activation of the flounder liver-type antifreeze protein gene (Apweiler et al., 2004).
- ***POLG2:*** This gene encodes a DNA repair and also DNA replication protein (Apweiler et al., 2004). This protein enhances DNA binding and promotes processive DNA synthesis (Maglott et al., 2005).
- ***RFC2_HUMAN, RFC3_HUMAN, RFC4_HUMAN and RFC5_HUMAN***: These genes encode a member of the activator 1 small subunits family. The elongation of primed DNA templates by DNA polymerase δ and ∈ requires the action of the accessory proteins, proliferating cell nuclear antigen (PCNA) and replication factor C (RFC). Replication factor C, also called activator 1, is a protein complex consisting of five distinct subunits. The core complex possesses DNA-dependent ATPase activity, which was found to be stimulated by PCNA in an *in vitro* system (Maglott et al., 2005).
- ***RFA1_HUMAN, RFA2_HUMAN and RFA3_HUMAN***: They play an essential role in several cellular processes in DNA metabolism including replication, recombination and DNA repair. They bind and subsequently stabilize single-stranded DNA intermediate and thus prevent complementary DNA from reannealing. They function as components of the alternative replication protein A complex (aRPA). aRPA binds single-stranded DNA and probably plays a role in DNA repair; it does not support chromosomal DNA replication and cell cycle progression through S-phase. *In vitro*, aRPA cannot promote efficient priming by DNA polymerase alpha but supports DNA polymerase delta synthesis in the presence of PCNA and replication factor C (RFC), the dual incision/excision reaction of NER and RAD51-dependent strand exchange (Apweiler et al., 2004).

## Conclusions

Measuring oxidative DNA lesions at the cellular level is a challenging task since the chosen methodology should be sensitive enough to measure using a few μg of DNA less than one modification per million DNA bases. In addition, the possibility to artefactually produce such lesions during for example DNA isolation increases the difficulty to obtain reliable results. Thus, effort should be made to compare different and independent experimental approaches in order to better assess the formation and biological consequences of DNA lesions. In the field of ionizing radiation, it is not only the chemical nature of the lesions that is important, but it is their localization in a cluster of damage i.e., MDS. This dense distribution of lesions increases their biological importance since the DNA repair systems are often challenged while processing of these clusters. In the future, effort should be also made to search for specific radiation-induced lesions. Since, following irradiation, several radicals could be produced in a close vicinity, one could imagine that such specific lesions could be generated by the recombination of two initially produced radicals. Such damage would have a very low probability to be generated by endogenous oxidative stress. Recently, it has been shown that such a lesion can be generated by the recombination of two initially produced radicals (Uvaydov et al., 2014).

Based on the current experimental tools one can envision that in the future the radiation-induced clustered DNA lesions will be more efficiently and more accurately detected at the level of human cells or tissues and will constitute the signature of this type of radiation, as it is the case for pyrimidine dimers with UV irradiation. In addition, by using various gene product markers one can hypothesize the development of biomarker libraries suggesting response to the specific type of stresses i.e., ionizing radiation, oxidative or replication stress.

### Conflict of interest statement

The authors declare that the research was conducted in the absence of any commercial or financial relationships that could be construed as a potential conflict of interest.
