# Supplementary material for: Stress-induced DNA damage biomarkers: applications and limitations
Source: Front Chem. 2015 Jun 2;3:35. doi: 10.3389/fchem.2015.00035 (PMC4451417; doi:10.3389/fchem.2015.00035)
Supplement: Supplementary file 1 [file DataSheet1.DOCX]

Supporting Information

**Stress-induced DNA damage biomarkers: Applications and limitations**

**Zacharenia Nikitaki, Christine E. Hellweg, Alexandros G. Georgakilas
and Jean-Luc Ravanat**

# SI Utilizing meta-analysis tools in order to reveal potent and existent DNA damage biomarkers

We explain here the steps followed in order to retrieve our results. By our first scan in the literature (please see below), **Table II** is created. With **Table II,** we suggest genes that their products could serve as biomarkers in general for the confirmation and quantitative assessment of existence of DNA damage when the origin of the damage is known i.e. ionizing radiation, oxidative or replication stress.

## Creation of Table II:

An example for retrieving ‘replication stress’ related biomarkers:

We begin with GLAD4U.

### GLAD4U

[GLAD4U](http://bioinfo.vanderbilt.edu/glad4u/index.php): (Gene List Automatically Derived For You) a web-based gene retrieval and prioritization tool from PubMed literature. GLAD4U takes advantage of existing resources of the NCBI to ensure computational efficiency [[2](#_ENREF_2)]. Firstly it collects the relative to the *query* publications, and then it keeps only those that contain (human) genes. For the prioritization of genes the gene-to-publication link table provided by Entrez-Gene is utilized. This table contains approx. 3x10^4^ human genes associated with approx. 3x10^5^ publications. For prioritization a hypergeometric test is performed. The result is given either as a list (from the more relevant to the less), either classified on tables according to biological processes, cellular components and molecular functions. The score for prioritization is given as the negative logarithm of the hypergeometric p-value.

### GLAD4U results:

As quoted in main text and in order to create the REPLICATION STRESS group of genes we put in GLAD4U the relative queries “DNA repair and replication stress”, “replication stress and DNA repair” and “replication stress and DNA damage response”. We present below what happens if we run the first query:

| GLAD4U - Vanderbilt University |
| --- |
| Department of Biomedical Informatics - <http://bioinfo.vanderbilt.edu/glad4u> |
|  |

[**Home**](http://bioinfo.vanderbilt.edu/glad4u/index.html) **|** [**News/Updates**](http://bioinfo.vanderbilt.edu/glad4u/outputs/lu7gr9sebma9i8jgue8tacjf70/lu7gr9sebma9i8jgue8tacjf70_results_1.html) **|** [**Documentation**](http://bioinfo.vanderbilt.edu/glad4u/outputs/lu7gr9sebma9i8jgue8tacjf70/lu7gr9sebma9i8jgue8tacjf70_results_1.html) **|** [**Contact Us**](http://bioinfo.vanderbilt.edu/glad4u/outputs/lu7gr9sebma9i8jgue8tacjf70/lu7gr9sebma9i8jgue8tacjf70_results_1.html)**
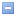
**  **Search** 
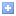
                 Ticket number: **lu7gr9sebma9i8jgue8tacjf70**

**Summary** [
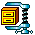
](http://bioinfo.vanderbilt.edu/glad4u/outputs/lu7gr9sebma9i8jgue8tacjf70/lu7gr9sebma9i8jgue8tacjf70_results.tar.gz) [
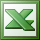
](http://bioinfo.vanderbilt.edu/glad4u/outputs/lu7gr9sebma9i8jgue8tacjf70/lu7gr9sebma9i8jgue8tacjf70_results.csv) [
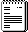
](http://bioinfo.vanderbilt.edu/glad4u/outputs/lu7gr9sebma9i8jgue8tacjf70/lu7gr9sebma9i8jgue8tacjf70_gene.txt) 
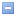

                **Generated on**: January 4, 2015
                **Query**: DNA repair and replication stress (Parameters used: threshold of 0.01, search only human genes, 100 genes per page, 10 publications per gene, 5 page links per page
                **Number of publications retrieved**: 1,123
                **Number of publications containing gene information (among the 1,123)**: 219
                **Number of genes in these 219 publications**: 351
                **Number of genes after the score threshold**: 88 [
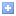
](http://bioinfo.vanderbilt.edu/glad4u/outputs/lu7gr9sebma9i8jgue8tacjf70/lu7gr9sebma9i8jgue8tacjf70_results_1.html)

**Send data to** [**Functional Enrichment Analysis**](http://bioinfo.vanderbilt.edu/glad4u/outputs/lu7gr9sebma9i8jgue8tacjf70/lu7gr9sebma9i8jgue8tacjf70_results_1.html) (opens a new window)

**Visualize genes in** [**a protein-protein interaction network**](http://bioinfo.vanderbilt.edu/glad4u/outputs/lu7gr9sebma9i8jgue8tacjf70/lu7gr9sebma9i8jgue8tacjf70_results_1.html)

Genes identified in your query, from highest to lowest scores:
(all links will open in new windows)
Expand all publications *
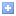
*

1. ATR - **ATR serine/threonine kinase**[Homo sapiens, Entrez-Gene ID:545]
   score: 70.4519, [go to Entrez-Gene page](http://www.ncbi.nlm.nih.gov/sites/entrez?cmd=search&db=gene&term=545), show the first 10 out of the 36 supporting publications 
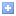


- Koganti S et al., STAT3 interrupts ATR-Chk1 signaling to allow oncovirus-mediated cell proliferation.Proc Natl Acad Sci U S A, 2014 Apr 1;111(13):4946-51 - [Abstract](http://www.ncbi.nlm.nih.gov/sites/entrez?cmd=search&db=pubmed&term=24639502)
- Couch FB et al., ATR phosphorylates SMARCAL1 to prevent replication fork collapse.Genes Dev, 2013 Jul 15;27(14):1610-23 - [Abstract](http://www.ncbi.nlm.nih.gov/sites/entrez?cmd=search&db=pubmed&term=23873943)
- Singh TR et al., ATR-dependent phosphorylation of FANCM at serine 1045 is essential for FANCM functions.Cancer Res, 2013 Jul 15;73(14):4300-10 - [Abstract](http://www.ncbi.nlm.nih.gov/sites/entrez?cmd=search&db=pubmed&term=23698467)
- Sowd GA et al., ATM and ATR activities maintain replication fork integrity during SV40 chromatin replication.PLoS Pathog, 2013;9(4):e1003283 - [Abstract](http://www.ncbi.nlm.nih.gov/sites/entrez?cmd=search&db=pubmed&term=23592994)
- Huntoon CJ et al., ATR inhibition broadly sensitizes ovarian cancer cells to chemotherapy independent of BRCA status.Cancer Res, 2013 Jun 15;73(12):3683-91 - [Abstract](http://www.ncbi.nlm.nih.gov/sites/entrez?cmd=search&db=pubmed&term=23548269)
- Reinson T et al., Engagement of the ATR-dependent DNA damage response at the human papillomavirus 18 replication centers during the initial amplification.J Virol, 2013 Jan;87(2):951-64 - [Abstract](http://www.ncbi.nlm.nih.gov/sites/entrez?cmd=search&db=pubmed&term=23135710)
- Sirbu BM et al., ATR-p53 restricts homologous recombination in response to replicative stress but does not limit DNA interstrand crosslink repair in lung cancer cells.PLoS One, 2011;6(8):e23053 - [Abstract](http://www.ncbi.nlm.nih.gov/sites/entrez?cmd=search&db=pubmed&term=21857991)
- Smits VA et al., Mechanisms of ATR-mediated checkpoint signalling.Front Biosci (Landmark Ed), 2010 Jun 1;15:840-53 - [Abstract](http://www.ncbi.nlm.nih.gov/sites/entrez?cmd=search&db=pubmed&term=20515729)
- Lovejoy CA et al., Functional genomic screens identify CINP as a genome maintenance protein.Proc Natl Acad Sci U S A, 2009 Nov 17;106(46):19304-9 - [Abstract](http://www.ncbi.nlm.nih.gov/sites/entrez?cmd=search&db=pubmed&term=19889979)
- Carson CT et al., Mislocalization of the MRN complex prevents ATR signaling during adenovirus infection.EMBO J, 2009 Mar 18;28(6):652-62 - [Abstract](http://www.ncbi.nlm.nih.gov/sites/entrez?cmd=search&db=pubmed&term=19197236)
- [see all supporting publications in PubMed](http://www.ncbi.nlm.nih.gov/pubmed/24639502,23873943,23698467,23592994,23548269,23135710,21857991,20515729,19889979,19197236,19004803,18995830,18931676,18283122,18003706,17638878,17616665,17384638,17376433,17030982,16757521,16474843,16431910,16260606,15539948,15485898,15282542,15050919,14742437,14724280,12791985,11721054,11673449,11418864,11163154,11114888)

In order to make **SI Table 1a** we combine the .tsv (tab separated values) files that arise from

*Summary third button*as shown from the original procedure: **Summary** [
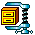
](http://bioinfo.vanderbilt.edu/glad4u/outputs/lu7gr9sebma9i8jgue8tacjf70/lu7gr9sebma9i8jgue8tacjf70_results.tar.gz) [
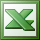
](http://bioinfo.vanderbilt.edu/glad4u/outputs/lu7gr9sebma9i8jgue8tacjf70/lu7gr9sebma9i8jgue8tacjf70_results.csv) [
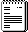
](http://bioinfo.vanderbilt.edu/glad4u/outputs/lu7gr9sebma9i8jgue8tacjf70/lu7gr9sebma9i8jgue8tacjf70_gene.txt)  and [**Functional Enrichment Analysis**](http://bioinfo.vanderbilt.edu/glad4u/outputs/lu7gr9sebma9i8jgue8tacjf70/lu7gr9sebma9i8jgue8tacjf70_results_1.html)**>**[Export TSV Only](http://bioinfo.vanderbilt.edu/webgestalt/htdocs/export.php?timestamp=1430414457) in an excel sheet.

We keep only the genes with score higher than 10.

By the same way **SI Table 1b** and **SI Table 1c** are created.

| **SI Table 1a** | |  | **SI Table 1b** | |  | **SI Table 1c** | |
| --- | --- | --- | --- | --- | --- | --- | --- |
|  | |  |  | |  |  | |
| **Query: “DNA repair and  replication stress”** | |  | **Query: “replication stress   and DNA repair”** | |  | **Query: “replication stress  and DNA damage response** | |
| **score** | **Gene** |  | **score** | **Gene** |  | **score** | **Gene** |
| 70.4519 | *ATR* |  | 70.4519 | *ATR* |  | 113.550027 | *ATR* |
| 26.893342 | *ATRIP* |  | 26.893342 | *ATRIP* |  | 48.339535 | *CHEK1* |
| 22.541925 | *ATM* |  | 22.541925 | *ATM* |  | 42.519784 | *ATM* |
| 16.968437 | *H2AFX* |  | 16.968437 | *H2AFX* |  | 32.409262 | *CLSPN* |
| 16.757653 | *RPA2* |  | 16.757653 | *RPA2* |  | 28.960509 | *ATRIP* |
| 14.280454 | *BLM* |  | 14.280454 | *BLM* |  | 23.842135 | *RPA1* |
| 13.741887 | *WRN* |  | 13.741887 | *WRN* |  | 20.822734 | *RPA2* |
| 12.281905 | *RPA1* |  | 12.281905 | *RPA1* |  | 15.62232 | *SMARCAL1* |
| 11.972673 | *RAD17* |  | 11.972673 | *RAD17* |  | 14.036702 | *RAD17* |
| 10.804183 | *CHEK1* |  | 10.804183 | *CHEK1* |  | 13.855259 | *BLM* |
| 10.324581 | *FANCM* |  | 10.324581 | *FANCM* |  | 13.317429 | *WRN* |
|  |  |  |  |  |  | 12.777728 | *H2AFX* |
|  |  |  |  |  |  | 11.447121 | *CDC25A* |
|  |  |  |  |  |  | 10.545647 | *RAD9A* |
|  |  |  |  |  |  | 10.253159 | *NBN* |

### Unifying data

After unifying the above three tables, **SI Table 1d** is produced.

Then, we perform again the prioritization according to the score and we delete the duplicates.

**SI Table 1e** is the resulted table for ‘Replication Stress’ part of **Table II**.

| **SI Table 1d** | |  | **SI Table 1e** | |
| --- | --- | --- | --- | --- |
| **REPLICATION STRESS** | |  | **REPLICATION STRESS** | |
| **score** | **Gene** |  | **score** | **Gene** |
| 113.550027 | *ATR* |  | 113.550027 | *ATR* |
| 48.339535 | *CHEK1* |  | 48.339535 | *CHEK1* |
| 42.519784 | *ATM* |  | 42.519784 | *ATM* |
| 32.409262 | *CLSPN* |  | 32.409262 | *CLSPN* |
| 28.960509 | *ATRIP* |  | 28.960509 | *ATRIP* |
| 23.842135 | *RPA1* |  | 23.842135 | *RPA1* |
| 20.822734 | *RPA2* |  | 20.822734 | *RPA2* |
| 15.62232 | *SMARCAL1* |  | 16.968437 | *H2AFX* |
| 14.036702 | *RAD17* |  | 15.62232 | *SMARCAL1* |
| 13.855259 | *BLM* |  | 14.280454 | *BLM* |
| 13.317429 | *WRN* |  | 14.036702 | *RAD17* |
| 12.777728 | *H2AFX* |  | 13.741887 | *WRN* |
| 11.447121 | *CDC25A* |  | 11.447121 | *CDC25A* |
| 10.545647 | *RAD9A* |  | 10.545647 | *RAD9A* |
| 10.253159 | *NBN* |  | 10.324581 | *FANCM* |
| 70.4519 | *ATR* |  | 10.253159 | *NBN* |
| 26.893342 | *ATRIP* |  |  |  |
| 22.541925 | *ATM* |  |  |  |
| 16.968437 | *H2AFX* |  |  |  |
| 16.757653 | *RPA2* |  |  |  |
| 14.280454 | *BLM* |  |  |  |
| 13.741887 | *WRN* |  |  |  |
| 12.281905 | *RPA1* |  |  |  |
| 11.972673 | *RAD17* |  |  |  |
| 10.804183 | *CHEK1* |  |  |  |
| 10.324581 | *FANCM* |  |  |  |
|  |  |  |  |  |

### Related publications

If the reader wishes to have access to all publications that are related to abovementioned genes, could go to GLAD4U homepage, insert the ticket number provided above and then to press

*Expand all publications 
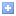
.*

### The other two sets of Table II data

By repeating with similar way the steps 1.1.2 and 1.1.3 the rest of **Table II** is created.

## Creation of Table I

In order to create **Table I** we follow another approach. We begin with the input “DNA repair” in AmiGO2.

### AmiGO2

[AmiGO2](http://amigo.geneontology.org/amigo) is a browser of Gene Ontology Consortium. The Gene Ontology (GO; http://www.geneontology.org) is a community-based bioinformatics resource that supplies information about gene product function using ontologies to represent biological knowledge. The Gene Ontology Consortium (GOC) has expanded areas of the ontology such as cilia-related terms, cell-cycle terms and multicellular organism processes. It is also implemented with new tools for generating ontology terms based on a set of logical rules making use of templates. The GOC web site summarizes developments and documentation, serving as a portal to GO data. User can perform GO enrichment analysis, and search the GO for terms, annotations to gene products, and associated metadata across multiple species using the AmiGO 2 browser [[4](#_ENREF_4)].

### “DNA repair” brunch of Table I

#### Applying filters step by step for “DNA repair AND ionizing radiation”

We show here how the results are being confined as we add extra filters.

If we begin with

<http://amigo.geneontology.org/amigo/medial_search?q=DNA+repair>

> Genes and gene products

It results with 12750 found entities.

Then we add the first filter

> taxon = homo sapiens 🡪at this moment we have 867 genes

Then one more filter

> Direct annotation = DNA repair 🡪590 genes

And one more

> Direct annotation = Response to ionizing radiation 🡪14 genes

Now we remove the above filter and add this one

> Direct annotation = Cellular response to ionizing radiation 🡪2 genes, but these two are already included in above mentioned 14 genes.

#### Applying filters for “oxidative stress” and also for “replication stress”

By this first screen we have a first estimation to the term

DNA repair AND ionizing radiation.

We repeat 1.2.2.1 steps also for

1. “Oxidative stress” and b) “replication stress”.

### “DNA damage response” brunch of Table I

We run the steps explained above (1.2.2) for DNA damage response

For a) ionizing radiation, b) replication stress and c) replication stress.

### Exclusion of common genes among the categories

At this time we have six times run the process.

We have results for:

1. DNA repair ∩ ionizing radiation
2. DNA repair∩ oxidative stress
3. DNA repair ∩ replication stress
4. DNA damage response ∩ ionizing radiation
5. DNA damage response ∩ oxidative stress
6. DNA damage response ∩ replication stress

Now is the time to unify 1) & 3) & the corresponding column from table II (IONIZING RADIATION).

Accordingly: 2) & 4) &the corresponding column from table II (OXIDATIVE STRESS).

And also: 3) & 6) & the corresponding column from table II (REPLICATION STRESS).

For each one of our new sets we delete the duplicates.

And finally we are only interested in UNIQUE genes. So we use BioVenn [[1](#_ENREF_1)] and for each set we keep only the genes that are not common with genes of any of the other two sets.

[BioVenn:](http://www.cmbi.ru.nl/cdd/biovenn/) As it is analytically explained in its home page, it is a web application for the comparison and visualization of biological lists using area-proportional Venn diagrams**.** It accepts as inputs up to 3 lists (x,y,z) of data and compares for common terms. It calculates the number of co-existence for each combination { x∩(y∪z)', y∩(x∪z)', z∩(x∪y)', x∩y∩z', x∩z∩y', y∩z∩x', x∩y∩z} and makes the representation. The user can retrieve the subsets that are created.

We also have to refer that for this step one can follow many other alternative approaches instead of BioVenn.

**This is the end of automated treating.**

After all these steps, manual treatment was performed as it is described in the main text.

# One step further: Testing our results by STRING v.10

Aiming to enhance the significance of our results towards the ‘uniqueness’of the biomarkers found in each category, we used our data of **Table I** as input into STRING v.10, in order to investigate if there are any interactions among the genes through the three classifications. The results based on experimental evidence show no interaction among the proteins of each group with another (no intergroup association).

## STRING v.10

STRING database (Search Tool for the Retrieval of Interacting Genes*/*Proteins) (http://string-db.org) provides a critical representation of protein–protein interactions, including direct (physical) as well as indirect (functional) associations [[3](#_ENREF_3)].

Parameters:

- Organism: homo sapiens
- Active prediction methods: only experiments (among the available that were neighborhood, gene fusion, co-occurrence, experiments, databases, textmining)
- Level of confidence: 0.700


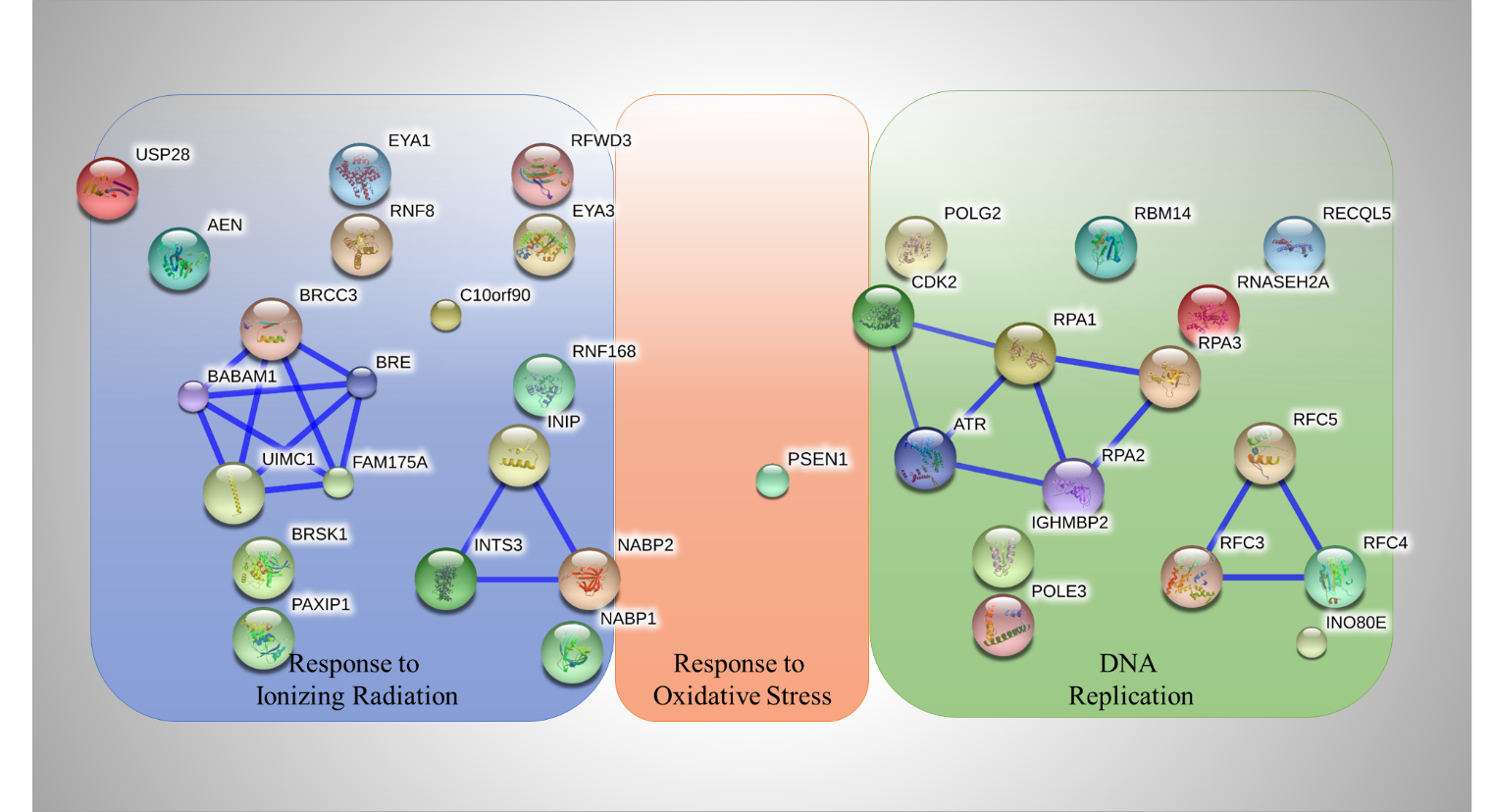


Fig. 1S: Protein-protein interaction network representation using the latest version of STRING database V.10, showing lack of interaction between the gene products of each classification of **Table I** based on experimental evidence. This result further supports the idea of 'uniqueness' for our biomarkers in **Table I** and for each type of stress i.e. ionizing radiation, oxidative and replication stress.
